# Discordant CSF/plasma HIV-1 RNA in patients with unexplained low-level viraemia

**DOI:** 10.1007/s13365-016-0448-1

**Published:** 2016-05-18

**Authors:** Sam Nightingale, Anna Maria Geretti, Apostolos Beloukas, Martin Fisher, Alan Winston, Laura Else, Mark Nelson, Stephen Taylor, Andrew Ustianowski, Jonathan Ainsworth, Richard Gilson, Lewis Haddow, Edmund Ong, Victoria Watson, Clifford Leen, Jane Minton, Frank Post, Munir Pirmohamed, Tom Solomon, Saye Khoo

**Affiliations:** 1 Institute of Infection and Global Health, University of Liverpool, The Ronald Ross Building, 8 West Derby Street, Liverpool, L69 7BE UK; 2 Department of Molecular and Clinical Pharmacology, University of Liverpool, Liverpool, UK; 3 Royal Liverpool and Broadgreen University Hospitals NHS Trust, Liverpool, UK; 4 Brighton and Sussex University Hospitals NHS Trust, Brighton, UK; 5 St Marys’ Hospital, Imperial College Healthcare NHS Trust, London, UK; 6 St Stephen’s AIDS Research Trust and Chelsea and Westminster Hospital NHS Foundation Trust, London, UK; 7 Birmingham Heartlands Hospital, Heart of England NHS Foundation Trust, Birmingham, UK; 8 North Manchester General Hospital, Pennine Acute Hospitals NHS Trust, Manchester, UK; 9 North Middlesex University Hospital NHS Trust, London, UK; 10 Research Department of Infection and Population Health, University College London, London, UK; 11 Victoria Royal Infirmary, Newcastle upon Tyne Hospitals NHS Trust, Newcastle, UK; 12 Royal Infirmary of Edinburgh, NHS Lothian, Edinburgh, UK; 13 Leeds General Infirmary, Leeds Teaching Hospitals NHS Trust, Leeds, UK; 14 Kings College Hospital NHS Foundation Trust, London, UK; 15 Walton Centre for Neurology and Neurosurgery, Liverpool, UK

**Keywords:** HIV, Cerebrospinal fluid, Antiretroviral agents, Drug resistance, Viral, Central nervous system

## Abstract

The central nervous system has been proposed as a sanctuary site where HIV can escape antiretroviral control and develop drug resistance. HIV-1 RNA can be at higher levels in CSF than plasma, termed CSF/plasma discordance. We aimed to examine whether discordance in CSF is associated with low level viraemia (LLV) in blood. In this MRC-funded multicentre study, we prospectively recruited patients with LLV, defined as one or more episode of unexplained plasma HIV-1 RNA within 12 months, and undertook CSF examination. Separately, we prospectively collected CSF from patients undergoing lumbar puncture for a clinical indication. Patients with durable suppression of viraemia and no evidence of CNS infection were identified as controls from this group. Factors associated with CSF/plasma HIV-1 discordance overall were examined. One hundred fifty-three patients were recruited across 13 sites; 40 with LLV and 113 undergoing clinical lumbar puncture. Seven of the 40 (18 %) patients with LLV had CSF/plasma discordance, which was significantly more than 0/43 (0 %) with durable suppression in blood from the clinical group (*p* = 0.005). Resistance associated mutations were shown in six CSF samples from discordant patients with LLV (one had insufficient sample for testing), which affected antiretroviral therapy at sampling in five. Overall discordance was present in 20/153 (13 %) and was associated with nadir CD4 but not antiretroviral concentrations in plasma or CSF. CSF/plasma discordance is observed in patients with LLV and is associated with antiretroviral resistance associated mutations in CSF. The implications for clinical practice require further investigation.

## Introduction

The central nervous system (CNS) has been proposed as a sanctuary site where HIV-1 replication may continue during stable antiretroviral therapy (ART). Concentrations of many antiretrovirals (ARVs) in cerebrospinal fluid (CSF) fall below the minimum inhibitory concentration for wild type virus, and there is large inter-individual variability in CSF penetration (Letendre et al. 2010b; Vissers et al. 2010; Best et al. 2012; Peluso et al. 2012; Ciccarelli et al. 2013; Cusini et al. 2013). ARVs may also have reduced activity in some of the cell populations that are susceptible to HIV infection in the CNS (Aquaro et al. 2002; Gray et al. 2013). HIV-1 RNA can be found at higher concentrations in CSF than in plasma in some patients (Eden et al. 2010; Rawson et al. 2012), termed CSF/plasma discordance, which may result in the compartmentalised detection of drug-resistant variants in CSF (Cunningham et al. 2000; Venturi et al. 2000; Strain et al. 2005).

Unexplained intermittent or persistent low-level viraemia (LLV) in plasma is a common clinical problem, occurring in up to a quarter of ART-treated patients (Doyle and Geretti 2012). The source of this virus is not clear and the clinical significance of LLV is debated (Doyle and Geretti 2012; Doyle et al. 2012). Whether the CNS can act as a sanctuary site for HIV-1 infection in these patients is not known. In this MRC-funded multicentre prospective study, we aimed to investigate the occurrence of CSF/plasma discordance in patients with LLV compared to patients with durable suppression of HIV-1 RNA in blood. We also aimed to investigate factors associated with CSF/plasma discordance, including ARV concentrations and resistance-associated mutations (RAMs) in the CSF.

## Methods

### Study population

We prospectively recruited HIV-1 positive adults (≥18 years) receiving ART from 13 centres in the UK. Written informed consent was obtained from all participants. The study was approved by the North Wales Research Ethics Committee (Central and East).

Patients were recruited in two groups. In the first group, patients with LLV underwent lumbar puncture (LP) for research purposes. LLV was defined as at least one episode of unexplained plasma HIV-1 RNA viraemia above detection threshold (40 copies/ml) within the previous 12 months. No upper limit was specified. We included patients with blips and persistent LLV. We excluded those with an alternative explanation for virological non-suppression such as adherence <95 % on self-report or recent commencement of ART. Recent modification of ART was not an exclusion.

It was not considered ethically justifiable to perform research LP on aviraemic controls without neurological symptoms; therefore, we prospectively recruited patients undergoing LP for a clinical indication and collected extra CSF at the time of LP. From this group, we identified patients with durable suppression of HIV-1 RNA detection limits (40 copies/ml) over the past year and no evidence of CNS infection to act as controls for those with LLV.

In a separate analysis, we examined factors associated with discordance in the two groups combined, including patients in the clinical group that were not durably suppressed or had evidence of CNS infection.

### Laboratory testing

Virology testing and quantification of drug concentrations were performed centrally at the University of Liverpool. CSF cell count, protein and glucose measurements and microbiological investigations were performed by routine methods at the centres of care. In the LLV group, CSF cell count was performed locally at the discretion of the treating physician.

### Virology testing

HIV-1 RNA was measured centrally in simultaneous plasma and CSF samples by the Abbott RealTime HIV-1 assay (Maidenhead, UK) with a lower limit of quantification of 40 copies/ml, as previously described (Garcia-Diaz et al. 2013). CSF/plasma discordance was defined as CSF HIV-1 RNA levels >0.5 log_10_ higher than those in plasma based on criteria from other studies (Rawson et al. 2012), and our own work showing raised CSF inflammatory cytokines at this degree of discordance (Nightingale et al. 2016). Discordant samples underwent Sanger (population) sequencing of reverse transcriptase (RT, amino acids 1 to 335), protease (amino acids 1 to 99) and integrase (amino acid 1 to 288) to detect RAMs, as previously described (Stockdale et al. 2015).

### Drug concentrations

ARV concentrations were determined in CSF and plasma by liquid chromatography-tandem mass spectrometry (LC-MS/MS). Drugs were extracted by protein precipitation (plasma) or directly injected (CSF). Standard curves were made up in artificial CSF (Harvard Apparatus, Holliston, MA, USA), and matrix effects assessed by post column injection. Assays were validated against repeated runs of quality controls (inter and intra-assay variability) and external quality assurance standards.

The revised CNS penetration effectiveness (CPE) score (2010) was calculated by assigning a predefined number of points to each component of the ART regimen, as proposed by Letendre et al. (Letendre et al. 2008, 2010a). Medication adherence was assessed using the modified medication adherence self report inventory (MASRI) questionnaire at the time of LP.

### Cognitive screening tools

In order to screen for issues related to cognitive impairment, patients with LLV underwent assessment with the following tools. Symptoms of cognitive difficulties were assessed by asking patients to answer whether they had problems with memory, reasoning and attention, as described by Simioni et al. (Simioni et al. 2010), and used in international guidelines (European AIDS Clinical Society 2014) [21] [21] [20] ^20^. Patients were considered to have cognitive symptoms when answering “yes, definitely” on at least one of the three questions. Patients were also screened for cognitive impairment using the International HIV Dementia scale (Zipursky et al. 2013). This scale uses three brief tests to assess motor speed, psychomotor speed and memory-recall. A score of less than ten has been considered potentially abnormal (Sacktor et al. 2005). Functional impairment was measured using the Instrumental Activities of Daily Living scale (Heaton et al. 2004; Mind exchange 2013). The maximum score of eight means no impairment in the following activities: telephoning, shopping, preparing food, housekeeping, laundry, travel, medications and finances. A score of seven or less indicates potential functional impairment. Mood disorders were assessed using the Hospital Anxiety and Depression scale, addressing depressive (HAD-D) and anxious (HAD-A) symptoms separately. Patients were considered depressed or anxious if the HAD-D or HAD-A subscale score was at least ten out of 21 (Savard et al. 1998, 1999). Cognitive testing was not performed in the clinical group as this could be confounded by intercurrent illness.

### Statistical analysis

Mann-Whitney *U* test was used to compare continuous non-parametric variables. Fisher’s exact and chi-squared tests were used for categorical data. The geometric mean of log_10_ ARV concentrations in plasma and CSF was compared between discordant and non-discordant patients with Student’s *t* test and also combined with time post dose in a multivariate logistic regression analysis using the forced entry method. A *p* value of 0.05 was used to determine statistical significance. When correlating CSF and plasma HIV-1 RNA, levels below detection were assigned an arbitrary mid-point value between zero and the 95 % detection rate. All analyses were performed using SPSS v22.

## Results

### Study population

A total of 153 ART-treated patients were recruited; 40 patients with LLV undergoing LP for research purposes and 113 with clinically indicated LP.

In the 40 patients with LLV, plasma HIV-1 RNA had been measured a median of five times (IQR 4, 6) in the previous 12 months. HIV-1 RNA had been detected >50 copies/ml on 117 of 198 occasions; median HIV-1 RNA levels during viraemic episodes was 92 copies/ml (IQR 59, 179). Eight patients (20 %) reported cognitive symptoms on questionnaire. One patient (3 %) had a score below ten on the International HIV Dementia scale and one other patient had a score below eight on the Instrumental Activities of Daily Living scale. Ten patients (25 %) were anxious and two patients (6 %) were depressed according to the Hospital Anxiety and Depression scale.

In the clinical group, 113 patients underwent LP to investigate cognitive symptoms (*n* = 50), headache (*n* = 20), suspected CNS infection or malignancy (*n* = 18), follow-up of a previous CNS infection (*n* = 8), abnormal neurological examination (*n* = 7), seizure (*n* = 5), neuropathy/radiculopathy (*n* = 2) and others (*n* = 3). On the basis of clinical findings and results of LP CNS infection was excluded in 92/113 patients (81 %). Where CNS infection was proven or probable, the diagnoses were neurosyphilis (*n* = 4), cryptococcal meningitis (*n* = 2), cryptococcal immune reconstitution inflammatory syndrome (*n* = 2), herpes simplex virus type-2 meningitis (*n* = 1), viral radiculopathy (*n* = 1), Ebstein-Barr virus in CSF of uncertain significance (*n* = 4) and no diagnosis (*n* = 7). Details of alternative diagnoses in those without CNS infection were not recorded; symptoms were cognitive (*n* = 43), headache (*n* = 16), suspected CNS infection or malignancy (*n* = 12), follow-up of a previous CNS infection (*n* = 4), abnormal neurological examination (*n* = 7), seizure (*n* = 5), neuropathy/radiculopathy (*n* = 2) and others (*n* = 3). In this group, 43/113 (38 %) patients had durable HIV-1 RNA suppression for 12 months and no CNS infection. The remaining 70 either had CNS infection (*n* = 21), were not suppressed (*n* = 26) or were not durably suppressed (*n* = 23).

### CSF/plasma HIV-1 RNA discordance

**Fig. 1:**
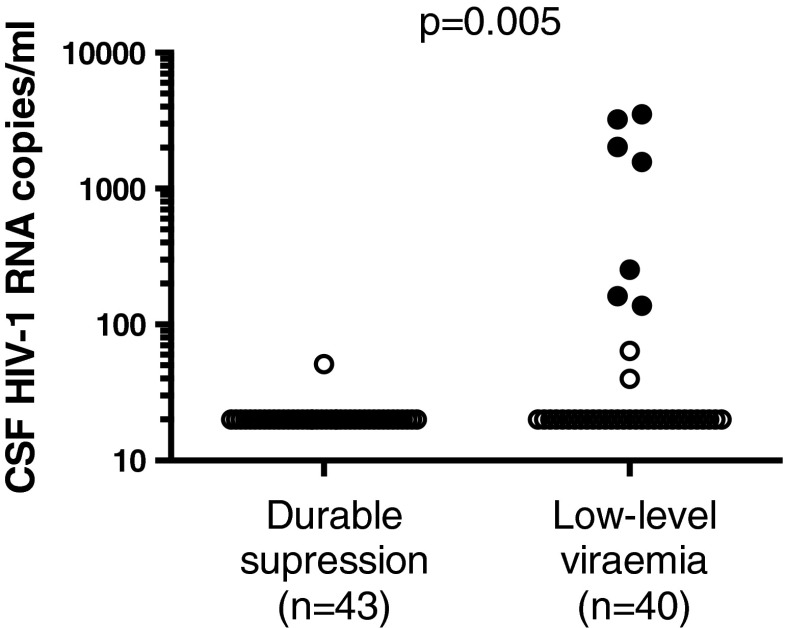
CSF HIV-1 RNA in 40 patients with intermittent or persistent low-level plasma HIV-1 RNA detection in the last year versus 43 patients from a clinical cohort with durable HIV-1 RNA suppression in the last year. *Filled circles* represent discordant samples

CSF/plasma discordance was found in 7/40 (18 %) patients with LLV. None of the 43 patients in the clinical group with durable suppression had discordance. The difference in rate of discordance between patients with LLV (18 %) and durable suppression (0 %) was statistically significant, *p* = 0.005 (Fig. 1). In patients with LLV, there was no difference in magnitude or frequency of HIV-1 RNA detection over the previous 12 months between those with (*n* = 7) and those without (*n* = 33) discordance. There were no significant differences in the rate cognitive symptoms, International HIV Dementia Scale results, functional impairment on the Instrumental Activities of Daily Living scale, or tests of anxiety and depression. There was a trend to more frequent raltegravir use in patients with LLV compared to durable suppression (11/40 versus 19/133, *p* = 0.060), which likely reflects use of integrase inhibitors to intensify ART in patients with viral persistence.

In the clinical group, discordance was found in 13/113 (12 %) patients. All discordant patients in this group were either not suppressed in plasma, not durably suppressed in plasma or had CNS infection. When combined with the LLV group, the rate of discordance overall was 20/153 (13 %). Overall HIV-1 RNA levels in plasma and CSF were directly correlated (Spearman *r* = 0.51; *p* < 0.001). HIV-1 RNA levels were <50 copies/ml in 113/153 (74 %) plasma samples and 115/153 (75 %) CSF samples. In discordant patients median HIV-1 RNA levels were 501 copies/ml (IQR 210, 2016) in CSF versus <40 copies/ml (IQR <40,<40) in plasma. Differences in measured variables among patients with and without CSF/plasma discordance are shown in Table 1. Patients with discordance showed a lower nadir (but not current) CD4 cell count and a higher CSF white cell count. Lower rates of discordance were observed in white MSM compared with black heterosexuals, which may reflect the higher nadir CD4 cell count in this group (median 173 versus 110 cells/mm^3^, *p* = 0.029).

**Table 1:** Univariate analysis of factors associated with discordant HIV-1 RNA levels in paired CSF and plasma samples

	CSF/plasma discordance		*p* value
	Yes (*n* = 20)	No (*n* = 133)	
Age, median years (IQR)	46 (43, 50)	46 (41, 54)	0.913
Gender male, *n* (%)	15 (75)	117 (88)	0.156
Risk group/ethnicity, *n* (%)			0.007
White MSM	7 (35)	80 (60)	
Black heterosexual	8 (40)	17 (13)	
Other	5 (25)	36 (27)	
CD4, median cells/mm^3^ (IQR)			
Nadir	32 (21, 256)	159 (49, 281)	0.030
Current	374 (190, 613)	464 (310, 707)	0.163
Years since HIV diagnosis, median (IQR)	10 (7, 16)	9 (5, 16)	0.833
ART at sampling, *n* (%)			0.178
PI/r based	14 (70)	74 (55)	
NNRTI based	3 (15)	47 (35)	
Other	3 (15)	12 (9)	
CPE, median (IQR)	7 (7, 10)	7 (6, 8)	0.250
Self reported adherence <95 %, *n* (%)	2 (10)	12 (9)	1.000
CSF WCC median cells/mm^3^ (IQR)	11 (<1, 21)	<1 (<1, 3)	0.018
CNS infection, *n* (%)	3 (15)	18 (14)	0.740

Risk group and ethnicity were combined due to co-linearity; 89 % of MSM were white. CSF white cell count was tested in 24 (60 %) of LLV patients and all patients in the clinical cohort

*MSM* men who have sex with men, *IVDU* intravenous drug user, *IQR* interquartile range, *PI/r* ritonavir-boosted protease inhibitor, *NNRTI* non-nucleoside reverse transcriptase inhibitor, *NRTI* nucleoside/tide reverse transcriptase inhibitor, *RAL* raltegravir, *MVC* maraviroc, *CPE* CNS penetration effectiveness score 2010

### CSF resistance

CSF samples from 6/7 discordant patients with LLV underwent sequencing for the detection of HIV-1 RAMs (one patient had insufficient CSF for resistance testing). All six samples tested showed RAMs; this affected one or more of the ARVs taken at the time of sampling in 5/6 patients (Table 2). At least one RAM had been previously identified in plasma in all six patients.

**Table 2:** CSF ARV resistance associated mutations (RAMs) in 20 patients with CSF/plasma discordance

HIV-1 RNA copies/ml				CSF resistance associated mutations			
Patient ID	Plasma	CSF	ART at sampling	PI	NRTI	NNRTI	INT
Low-level viraemia group							
1	<40	138	TDF/FTC/DRV/r	–	***M184I***	–	–
2	<40	254	EFZ/TDF/FTC	Insufficient sample			
3	48	2028	DRV/r/TDF/RAL	None	***D67N***, ***K70R***, ***L74V***, ***M184V***, ***T215Y***, ***K219E***	*L100IL*, *K103KN*	**T66I, Y143C**
4	78	3234	DRV/r/MVC/ETR	None	*A62V*, *K65R*, *M184V*	None	–
5	88	1569	TDF/FTC/DRV/r	None	***M184I***	None	–
6	<40	162	TDF/FTC/DRV/r	V82A	***D67N***, ***M184V***, ***T215Y***, ***K219Q***	V108I	–
7	258	3518	ABC/3TC/DRV/r	None	**L74LV**, ***M184V***	None	–
Clinical cohort							
8	40	1038	TDF/FTC/DRV/r	None	Incomplete testing		
9	52	1231	TDF/FTC/RAL/MVC	Did not amplify			
10	3443	13088	3TC/AZT/EFZ	–	None	**V108I**, **E138A**	
11	10817	63010	TDF/FTC/RAL	–	None	None	**L74I**
12	<40	400	ABC/3TC/DRV/r/RAL	Did not amplify			
13	<40	422	TDF/FTC/ATZ/r	–	**T69S, M184I**	–	–
14	<40	335	TDF/FTC/DRV/r	Did not amplify			
15	<40	162	3TC/AZT/DRV/r/MVC/ETR	Insufficient sample			
16	<40	129	DRV/r	Did not amplify			
17	<40	579	ATZ/r/TDF/FTC	**V82AV**	**M184V**, **T215I**	–	–
18	<40	1981	DRV/r/RAL/MVC/3TC	None	***M184V***	V108I	***N155H***
19	<40	195	EFZ/TDF/FTC	Did not amplify			
20	<40	405	LPV/r/AZT/RAL	None	None	None	**N155H**

RAMs in bold are to ARVs taken at the time of sampling. RAMs in italics have been previously detected in plasma. Patient 9 showed CCR5 tropic virus in CSF

*PI* protease inhibitor, *NRTI* nucleoside/tide reverse transcriptase inhibitor, *NNRTI* non-nucleoside reverse transcriptase inhibitor, *INT* integrase inhibitor, *ATZ* atazanavir, *DRV* darunavir, *LPV* lopinavir, *ETV* etravirine, *MVC* maraviroc, *NVP* nevirapine, *RAL* raltegravir, *RPV* rilpivarine, *r* ritonavir, *EFZ* efavirenz, *TDF* tenofovir, *FTC* emtricitabine, *3TC* lamivudine, *AZT* zidovudine

In the clinical group, 12/13 CSF samples from discordant patients underwent sequencing (one patient had insufficient CSF for resistance testing). Five CSF samples did not amplify despite repeated attempts and one sample only amplified for the protease gene. Of the six CSF samples successfully tested, all showed RAMs; this affected one or more of the ARVs taken at the time of sampling in all six patients (Table 2). CSF RAMs had been previously identified in plasma in one patient (patient 18).

Resistance testing of plasma virus was attempted in patients 7, 10 and 11. Plasma virus from patients 7 and 10 did not amplify. Plasma from patient 11 showed no NRTI or NNRTI RAMs; the integrase gene did not amplify.

### Plasma and CSF pharmacokinetics

ARV concentrations in plasma and CSF are shown in Table 3. No significant differences were observed between those with or without discordance for any ARVs tested in either CSF or plasma. There were also no significant differences in ARV concentrations between those with LLV versus durable suppression. No associations were observed with CPE score or ART use, either for individual ARVs or drug class of regimen (Table 1).

**Table 3:** Antiretroviral concentrations in CSF and plasma

	*n*	Drug concentration (ng/ml)	
		Geo mean	95 % CI
Plasma			
Tenofovir	62	89.5	73.1–110.7
Emtricitabine	65	187.9	136.5–264.9
Lamivudine	24	383.7	225.4–688.7
Darunavir	55	2971.7	2393.3–3715.4
Atazanavir	19	729.5	418.8–1339.7
Lopinavir	3	8570.4	7798.3–9440.6
Ritonavir	15	116.1	55.7–277.3
Efavirenz	27	1741.8	1349.0–2269.9
Nevirapine	10	4226.7	2691.5–6807.7
Etravirine	4	269.2	64.4–1836.5
Rilpivarine	4	68.1	36.0–144.2
Maraviroc	8	172.2	88.9–367.3
Raltegravir	11	413.9	174.1–1135.0
CSF			
Darunavir	54	35.2	27.8–45.4
Atazanavir	20	6.4	4.1–11.5
Lopinavir	3	9.6	4.1–37.3
Efavirenz	26	14.7	11.4–19.4
Nevirapine	12	1559.6	1127.2–2197.9
Maraviroc	9	6.5	2.75–31.6
Raltegravir	14	25.9	18.1–38.7

Geometric mean and 95 % confidence interval for drug concentrations in plasma and CSF. No significant associations were observed with CSF/plasma discordance

## Discussion

We observed CSF/plasma discordance in 18 % of patients undergoing a research-based LP for intermittent or continuous LLV. No discordance was observed in patients with durably suppressed plasma viral load undergoing clinically indicated LP. This suggests that a significant proportion of HIV-positive patients with LLV despite ART may have suboptimal control of virus replication within the CNS. CSF examination of discordant patients with LLV revealed RAMs in all samples tested, which related to ART taken at the time of sampling in all but one. Of note, in these patients, plasma viraemia was generally too low to perform resistance testing in blood.

One interpretation of these findings could be that ongoing replication of HIV in a CNS sanctuary site results in seeding to plasma leading to LLV. Alternatively, the observation of LLV and CSF/plasma discordance may represent two coinciding aspects of overall suboptimal virological suppression. It is of interest that in all cases at least part of the resistance detected in CSF virus was consistent with the previous history of failure and older resistance tests in plasma. This, combined with the observed association of discordance with low nadir CD4, suggests that advanced immune suppression may allow the establishment of a viral reservoir in the CNS that may be only partially responsive to ART. This virus may then continue to evolve and develop resistance to some ARVs, for example raltegravir, and thus become progressively less susceptible to suppression than virus in peripheral blood. LLV with a genetically different virus may originate from a similar process of incomplete suppression at other sites such as lymphoid tissue, where drug penetration and activity may also vary (Fletcher et al. 2014). Intermittent reseeding of virus into the CNS during periods of systemic replication due to another cause (e.g. non-declared poor adherence) may also establish compartmentalised CNS infection (Gisslen et al. 2005; Eden et al. 2010). Regardless of the origin of CSF virus, targeting LP to patients with unexplained blips or persistent LLV in plasma may represent a useful strategy in clinical practice and warrants further investigation. Altering ART based on the resistance profile of CSF variants has led to clinical improvement and decrease in CSF HIV-1 RNA in case series (Canestri et al. 2010; Peluso et al. 2012) and has been adopted by guidelines as best practice (Mind exchange 2013; Williams et al. 2014). In addition, these findings add to the evidence suggesting the CNS compartment must be considered in studies aiming to eradicate HIV from the periphery.

Screening for cognitive impairment in the LLV group with a cognitive symptoms questionnaire and International HIV-Dementia Scale did not reveal associations with CSF/plasma discordance. The numbers are small and the screening methods used are insensitive for milder cognitive disorders (Haddow et al. 2013); nevertheless, these data suggest that discordant patients were relatively asymptomatic and cognitive deficits were not marked. Whether discordance in this group has implications for cognitive dysfunction in the longer term requires clarification in longitudinal studies.

CSF/plasma discordance was present in 12 % of patients undergoing clinically indicated LP. This rate of discordance is similar to other reports from clinical settings (Eden et al. 2010; Rawson et al. 2012). Studies where LP was performed for research purposes in the absence of clinical indications have reported lower rates of discordance (Heaton et al. 2010). CSF/plasma discordance overall was not associated with ART at sampling, CPE score or ARV concentrations in plasma or CSF, but was associated with nadir CD4 cell count. This suggests the presence of HIV in the CNS relates to a longer duration of viral replication and a lower immune status, consistent with the hypothesis of CNS viral persistence discussed above. In cross sectional studies, nadir CD4 has been consistently associated with cognitive impairment (Robertson et al. 2007; Heaton et al. 2010). It has not been clear whether this association is due to a legacy effect of CNS damage sustained prior to ART initiation or whether it represents an ongoing CNS inflammatory process set up during advanced immunosuppression. In our study, all discordant CSF samples successfully tested showed RAMs. Overall RAMs were demonstrated in 12 CSF samples including NRTI RAMs in eight of ten on NRTIs at sampling and raltegravir RAMs in all four patients on raltegravir at sampling. Other work on this cohort has demonstrated raised inflammatory cytokines in CSF of patients with discordance, even at relatively low levels (Nightingale et al. 2016).

There are a number of limitations to our study. Selecting controls from patients undergoing clinically indicated LP was not ideal; however, as clinical cohorts have shown higher rates of discordance than those undergoing LP for research purposes, this risked producing a false negative rather than false positive result. We assessed adherence by self-report which may not be reliable in all patients. Of note, there were no observed differences in measured plasma ARV concentrations between those with and without discordance suggesting that adherence was similar between groups, at least in terms of very recent ART use. Differences in the rate of viral decay between compartments in those recently starting or changing ART could have led us to identify some with discordance that did not have true compartmentalisation (Schnell et al. 2009). Discordant patients in the LLV group had been on ART for many years; however, recent ART modification was not an exclusion and could have contributed. In the clinical group, we did not routinely collect details of ART history and cannot exclude that this was the source of discordance in some patients. Our analysis of factors related to discordance involved grouping the LLV group with the more heterogeneous clinical group, some of which were not suppressed and/or had CNS infection. This group is reflective of patients undergoing CSF examination in the UK clinical practice and may be more transferable to the clinical environment. Indeed studies recruiting asymptomatic patients for research LP, such as the CHARTER cohort in the USA, found few cases of discordance despite sampling large patient populations (Heaton et al. 2010). Our ability to find associations of discordance with ARV concentrations in CSF and plasma was limited by few measurements for some drugs. In addition, ARV concentrations in CSF may not be the most important factor determining effectiveness of ART in the CNS (Calcagno et al. 2014), in particular ARV concentrations in CSF may not reflect levels within perivascular macrophages, the critical target of HIV in the brain (McArthur et al. 2010). Our finding of no link between CPE score and discordance should also be interpreted with caution as our study was not designed to look for such an association and may be confounded by prescriber bias, whereby patients with CNS problems are more likely to receive an ART regimen with a higher CPE score (Garvey et al. 2011).
